# Toward achieving the WHO 2030 target in the mitigation of snakebite envenoming: the fundamental challenges in addressing the unmet needs

**DOI:** 10.1186/s41182-025-00754-0

**Published:** 2025-05-22

**Authors:** Swati Allen, Joy Kumar Chakma

**Affiliations:** Scientist C, 2Scientist F, Indian Council of Medical Research Department of Health Research, Faculty of Medical Research, AcSIR (Academy of Scientific and Innovative Research, an Institute of National Importance Established By an Act of Parliament), V. Ramalingaswami Bhawan, Ansari Nagar East, New Delhi, 110029 India

**Keywords:** Snakebite envenoming, Priority neglected tropical disease, Low- and middle- income countries, Prevention and control, Public health

## Abstract

Snakebite envenoming represents a significant public health challenge, particularly in regions where venomous snakes are prevalent. Globally, it has been estimated that every year 81,000–1,38,000 people die due to venomous snakebites along with enormous morbidity and physical disability to the survivors. Almost 70% of estimated global snakebite deaths are from South Asia Region. However, more than half of the global burden due to snakebite is alone from India with an estimated annual average of about 58,000 deaths. Thus, this is a significant public health problem for a developing country like India compared to the other Low- and Middle-Income Countries (LMICs). Considering the problem of snakebites in developing and tropical countries, which contributes almost 95% of the total snakebites of the world, the World Health Organization (WHO) has re-designated snakebite as a priority Neglected Tropical Disease (NTD) in 2017. However, there is a little more than a 5-year window left for reducing mortality and morbidity due to snakebite envenoming by 50%, in aligning with the WHO 2030 target. Thus, for achieving this target within the span of half a decade, for this decades-old problem, there is an urgent need to recognize the fundamental challenges for addressing the unmet needs and recognizing the opportunities.

The snakebite envenoming is a major public health problem in regions where venomous snakes are prevalent leading to significant mortality, morbidity and disability. Moreover, the victims of snakebite are mostly the rural and socioeconomically backward population. South Asia Region is a biodiversity hotspot for venomous snakes and contribute nearly 70% of estimated global snakebite deaths of about 81,000–1,38,000 every year from. However, more than half of the global burden of snakebite is alone from India with an estimated annual average of about 58,000 deaths resulting from the 3–4-million cases of snakebite envenoming each year [1]. India is having nearly 18% of the world population with almost 65% of India’s population lives in rural area [2] and about 55% are directly engaged in agricultural and associated endeavors [3]. Consequently, over half of our 1.4-billion populace [4] faces heightened vulnerability to snakebite incidents attributable to occupational exposure, outdoor activities, and suboptimal housing conditions. Of particular concern is the prevalent lack of awareness and education among these demographics on how to avoid snakebites with simple preventive and personal protective measures and what to if case, of bitten by snake leading to either improper treatment by local traditional healer or delay in seeking proper treatment thereby increasing the risk of complications. The delay in seeking treatment is also due to poor rural infrastructures, constrained financial resources coupled with stressed out and inadequate public healthcare infrastructure [5]. There is also significant numbers of snakebite happens in domestic animals and livestock with a case fatality rate of 47% leading to huge economic loss to the animal farmers in their livelihood [6]. Paradoxically, despite harboring the highest burden of snakebite incidents globally, India lacks precise epidemiologic data on Disability Adjusted Life Years (DALYs) attributable to snakebites [7]. Moreover, at this juncture, India is undergoing major epidemiologic transition and the country’s health system is facing a dual challenge in tackling the burden of communicable to non-communicable diseases. 

The crucial time has already started for fully rolling out the implementation plan 2025–2030 in line with the WHO Strategy for Prevention and Control of Snakebite Envenoming for achieving the 2030 target. To keep pace with the WHO Strategy, India has developed a comprehensive “National Action Plan for Prevention and Control of Snakebite Envenoming (NAP-SE)” aligning with the four strategic pillars envisaged by WHO and launched in March, 2024. This is a significant initiative toward mitigation of this many decades-old public health problem of mostly the rural poor and vulnerable population. The national action plan drawn the illustrative roadmap for successful implementation of the strategies based on the following four major pillars:

1. Governance and management of programme,
2. Intersectoral coordination and collaboration,
3. Advocacy and capacity building, and
4. Research and innovation

However, to achieve the WHO target within the given span of time, it is imperative to identify and analyze the fundamental challenges which are inherent within each pillar and the strategies for effectively addressing them in time. There is an intricate web of poor outcome determinants and barriers which are fundamentally inherent in the prevention and control of snakebite envenoming in India (Table) requiring urgent intervention toward achieving the WHO target of reducing the mortality and morbidity due to snakebite by 50% by 2030.

**Table:** 

Table illustrating the intricate web of poor outcome determinants and barriers which are fundamentally inherent in the prevention and control of snakebite envenoming in India	
Health sector	The inherent fundamental challenges
• Ensuring provision of Anti Snake Venom (ASV) at all health facilities	• Currently 1.5–2-million vials of ASV are manufactured in India which not adequate for ensuring the uninterrupted supply for the treatment of the 3–4-million cases of snakebite envenoming each year [1, 6] • In India, there are 23 medically important snakes including the Big-4 species which are responsible for the majority snakebites, However, there are also significant numbers of snakebites from the venomous snakes other than the Big-4, for which currently no ASV available [1, 6] • Lack of diagnostic tools lead to delayed diagnosis and initiation of treatment with ASV
• Strengthening surveillance of snakebite cases and deaths in humans	• The Government of India declared the snakebites’ cases and deaths a “Notifiable Disease” to mandatory report all suspected, probable snakebite cases and deaths by all government and private health facilities (including medical colleges) • However, there is no mechanism how the suspected, probable snakebite cases and deaths will be confirmed for actual mortality and morbidity statistics • Without the actual baseline and the gap between estimated and reported cases of snakebite and deaths, monitoring the progress of the program will be difficult for aligning the target during midterm evaluation
• Strengthening the emergency care services at district hospitals/CHC (community health center) including services for ambulance	• It is a longstanding unmet need for the country’s health system at the district/sub-district/block level in meeting the Indian Public Health Standard for providing the quality health care • Since, the victims of the snakebite are mostly rural and socioeconomically vulnerable population [1], the urgent attention is required to strengthen the CHC and also the PHC (primary health center) where snakebites are more prevalent to make the emergency services available, accessible and affordable to them • To ensure the appropriate management and timely referral of snakebite requiring advance care to higher center
• Capacity building by training of health professionals	• Time to time training of the doctors, nurses, and paramedics working in the CHC and PHC on snakebite treatment • Uniform adherence to the National Standard Treatment Guidelines for snakebite management in public and private health facilities • Prevention of the irrational intradermal testing of ASV leading to the wastage of the lifesaving ASV [6]
• Institutionalization of Regional Venom Centers (RVCs)	• This a very highly ambitious strategy and is the need of hour for mitigation of the venomous snakebite once for all not only in the direction of achieving the WHO 2023 but also in line with the honorable Indian prime minister vision of Viksit Bharat for achieving the “Zero Snake Bite” on or before 2047 • However, it lacks a comprehensive plan on how all these institutions will be brought together in one platform. Moreover, the leadership role is missing for the setting up of these RVCs for undertaking R & D (Research & Development) activities defined under these centers. The Premier biomedical research organization like the Indian Council of Medical Research could have been roped in for the leadership role
• Public–private partnership and intersectoral coordination	• More concerted and close coordination will require and the detailed mechanism for collaboration and or partnership of accomplishing the strategic requirement
Animal/agriculture health sector	• Currently 1.5–2-million vials of ASV are manufactured in India which not adequate for ensuring the uninterrupted supply for the treatment of the 3–4-million cases of human snakebite envenoming each year [1, 6]. Moreover, there are no baseline data on animal and livestock snakebites
• Ensuring provision of Anti Snake Venom at all veterinary facilities	
• Prevention of snakebites in livestock	• Close coordination and collaboration for intersectoral convergence between the Agricultural and Animal Husbandry/Veterinary Service departments
Wildlife and forest sector	• A holistic multipronged approach is require through the dynamics of humans, snakes and animals and environment which are intricate snakebite envenoming in human and animals for educating and creating awareness in the community level • Involvement of all stakeholders, such as the health and veterinary sectors, the Panchyati raj institutions and the local community
Social security	• Paying appropriate uniform compensation to all the victims of snakebite across all the States and Union Territories • Covering the snakebite treatment under the social security scheme of AB-PMJAY (Ayushman Bharat Pradhan Mantri Jan Arogya Yojona)

Although, the NAP-SE has been launched well within the time frame, the challenges (Table) lies in transitioning and implementing the strategies smoothly and timely within the existing health system, which is already stressed in dealing with the current situation of dual disease burden. Moreover, in providing quality health care services, the major unmet need in the country is inadequate health infrastructure in meeting the Indian Public Health Standard (IPHS), especially in the rural and vulnerable areas. Other than the health system, the multisectoral collaboration and convergence creating strong public–private partnerships require very close coordination, and a monitoring and evaluation mechanism is crucial for the success of the NAP-SE. In addition, key stakeholders like rural development and road transport also play a crucial role in not only improving the socioeconomic condition but also the health of the rural population and should have been part of the NAP-SE. Overall, NAP-SE has a clear roadmap with optimism; however, the journey has just started for reaching the ambitious target by 2030. Moreover, the success of reaching the destination will depend on overcoming the intricate challenges at the right time with robust implementation and monitoring mechanisms with close intersectoral collaboration and coordination for convergence, along with sustained efforts to educate and empower the communities, particularly in the vulnerable areas across the country. By doing so, NAP-SE has the potential to change the country’s scenario of snakebite envenoming and lead India in setting a global example in the mitigation of this decades-old public health problem.

## Data Availability

No datasets were generated or analysed during the current study.
